# An ancestry informative marker set for determining continental origin: validation and extension using human genome diversity panels

**DOI:** 10.1186/1471-2156-10-39

**Published:** 2009-07-24

**Authors:** Rami Nassir, Roman Kosoy, Chao Tian, Phoebe A White, Lesley M Butler, Gabriel Silva, Rick Kittles, Marta E Alarcon-Riquelme, Peter K Gregersen, John W Belmont, Francisco M De La Vega, Michael F Seldin

**Affiliations:** 1 Rowe Program in Human Genetics, Departments of Biochemistry and Medicine, University of California Davis, Davis, CA 95616, USA; 2 Applied Biosystems, Foster City, CA 94404, USA; 3 Department of Public Health Sciences, University of California Davis, Davis, CA 95616, USA; 4 Obras Sociales del Hermano Pedro, Antigua, Guatemala; 5 Section of Genetic Medicine, Department of Medicine, University of Chicago, Chicago, Illinois 60637, USA; 6 Department of Genetics and Pathology, Rudbeck Laboratory, Uppsala University, Uppsala, Sweden; 7 The Robert S. Boas Center for Genomics and Human Genetics, Feinstein Institute for Medical Research, North Shore LIJ Health System, Manhasset, NY 11030, USA; 8 Department of Molecular and Human Genetics, Baylor College of Medicine, Houston, TX 77030, USA

## Abstract

**Background:**

Case-control genetic studies of complex human diseases can be confounded by population stratification. This issue can be addressed using panels of ancestry informative markers (AIMs) that can provide substantial population substructure information. Previously, we described a panel of 128 SNP AIMs that were designed as a tool for ascertaining the origins of subjects from Europe, Sub-Saharan Africa, Americas, and East Asia.

**Results:**

In this study, genotypes from Human Genome Diversity Panel populations were used to further evaluate a 93 SNP AIM panel, a subset of the 128 AIMS set, for distinguishing continental origins. Using both model-based and relatively model-independent methods, we here confirm the ability of this AIM set to distinguish diverse population groups that were not previously evaluated. This study included multiple population groups from Oceana, South Asia, East Asia, Sub-Saharan Africa, North and South America, and Europe. In addition, the 93 AIM set provides population substructure information that can, for example, distinguish Arab and Ashkenazi from Northern European population groups and Pygmy from other Sub-Saharan African population groups.

**Conclusion:**

These data provide additional support for using the 93 AIM set to efficiently identify continental subject groups for genetic studies, to identify study population outliers, and to control for admixture in association studies.

## Background

As we and others have previously discussed, ancestry informative markers (AIMs) can be used as a tool to minimize bias due to population stratification in case-control association studies [1-4]. These AIMs are not necessary in genome-wide association studies (GWAS) since the data contains a wealth of SNP information that can define and control for population stratification [3]. However, AIMs may be particularly valuable for follow-up studies to confirm GWAS results or for focused candidate gene studies. These may include studies examining different continental populations as well as studies examining populations of mixed ancestry. Thus, it is timely to identify sets of AIMs that can be used to either pre-define subject groups or control for ancestry. Recently, our group has demonstrated the application of a set of SNP AIMs for discerning continental population information[4]. These studies using a total of 128 SNPs and subsets derived from this panel showed the ability of small sets of SNPs to separate a variety of self-identified subjects of European, Amerindian, East Asian, and sub-Saharan African ancestry. Using these SNPs we were able to provide admixture information for sub-Saharan African, European and Amerindian admixed populations, and perform structured association testing in the context of mixed or admixed population groups. In addition, these studies showed that a subset of 96 AIMs performed well in TaqMan^® ^assays, thus enabling potential wide application of these SNPs.

In the current study, we further examine the ability of a set of 93 AIMs to ascertain the ancestry of diverse population groups. This study was facilitated by the recent publically available Human Genome Diversity Panel (HGDP) genotypes[5] that include our 128 SNP AIM set. For the current study, we chose the set of 96 TaqMan optimized SNP AIMs that were the most informative AIMs from our previous study that had clear profiles in TaqMan assays[4]. Of these 96 AIMs, 93 (Additional File 1) had HGDP genotypes that passed quality filters (see Methods). These additional data allow the assessment of this SNP AIM set in Oceana populations and multiple additional African, South Asian, Amerindian, and European population groups. In addition, since our previous studies relied on several population groups (East Asian, South Asian and European) that were derived from collections in the United States, it was important to further validate the previous results using samples that were collected from specific countries of origin.

## Methods

### Populations studied

The individuals used in these studies include those from the HGDP, HapMap, the New York Cancer Project (NYCP) [6] and samples collected in the United States (Houston, Sacramento), Guatemala, Peru, Sweden. and West Africa. For the HGDP and HapMap the genotypes were available from online databases. For the other sample sets the genotyping was performed at Feinstein Institute for Medical Research (North Shore LIJ Health System) using Illumina 300 K array or using TaqMan assays as previously described [4]. Of the total of 1620 individual participant genotypes, 825 were included in our previous studies[4].

The previous genotypes included those from 128 European Americans, 88 African Americans, 60 CEPH Europeans (CEU), 56 Yoruban sub-Saharan Africans (YRI), 19 Bini sub-Saharan Africans, 23 Kanuri West Africans, 50 Mayan Amerindians, 26 Quechuan Amerindians, 29 Nahua Amerindians, 40 Mexican Americans (MAM), 26 Mexicans from Mexico City, 28 Puerto Rican Americans, 43 Chinese (CHB), 43 Chinese Americans, 43 Japanese (JPT), 3 Japanese Americans, 8 Vietnamese Americans, 1 Korean American, 45 Filipino Americans, 2 unspecified East Asian Americans, and 64 South Asian Indian Americans. The Maya-Kachiquel were Maya from the Kachiquel language group as previously described[7] and is from a collection distinct from the HGDP Maya group.

The additional subject sets in the current study included the following HGDP genotypes: Adygei (also known as Adyghe)(14 individuals), Balochi (15), Bantu from Kenya (12), Bantu from South Africa (8), Basque (13), Bedouin (47), Biaki Pygmy (32), Burusho (7), Cambodian (10), Columbian (7), Daur (10), Druze (43), Kalash (18), Lahu (8), Mandenka (24), Maya (13), Mbuti Pygmy (15), Melanesian (17), Mongolian (9), Mozabite (30), Palestinian (26), Papuan (16), Pima (11), Russian (13), San (7), Uygur (10), Yakut (15), Yi (10), and Yoruba (25). Other additional samples not previously studied included Ashkenazi Jewish (40), Swedish (40), Irish (40) and other European Americans (190) from the NYCP. Genotypes from the HGDP subjects were obtained from the NIH Laboratory of Neurogenetics .

For all subjects, blood cell samples were obtained according to protocols and informed-consent procedures approved by institutional review boards, and were labeled with an anonymous code number linked only to demographic information.

### Data Filters

SNPs and individual samples with less than 90% complete genotyping information from any data set were excluded from analyses. SNPs that showed extreme deviation from Hardy-Weinberg equilibrium (p < 0.00001) in individual population groups were also excluded from these analyses.

### Statistical Analyses

F_st _was determined using Genetix software[8] that applies the Weir and Cockerham algorithm[9]. Hardy-Weinberg equilibrium was determined using HelixTree 5.0.2 software (Golden Helix, Bozeman, MT, USA).

Population structure was examined using STRUCTURE v2.1[10,11] parameters and AIMs previously described[4]. Briefly, each analysis was performed without any prior population assignment and was performed at least 3 times with similar results using >200,000 replicates and >100,000 burn-in cycles under the admixture model. For all analyses reported, we used the "infer α" option with a separate α estimated for each population (where α is the Dirichlet parameter for degree of admixture). Runs were performed under the λ = 1 option where λ estimates the prior probability of the allele frequency and is based on the Dirichlet distribution of allele frequencies.

PCA was performed using the EIGENSTRAT statistical package[12].

## Results

### AIMs Show Increased Ability to Differentiate Between Continental Population Groups

Wright's F statistic, F_st_, was used as a common measure of population differentiation and calculates the inter-population compared to intra-population variation. Using the Weir and Cockerham algorithm[9] (see Methods) we compared the F_st _values of selected population groups between random marker sets and the 93 SNP AIMs. The studies included samples derived from HapMap [13,14], HGDP[5], and samples collected in the United States, Guatemala and Nigeria (see **Methods**). The random SNP F_st _values were obtained using three random non-overlapping sets of 3500 SNPs distributed over the autosomal genome (minimum of 50 kb distance between SNPs). The small differences in these triplicate independent samplings (mean SD for all paired F_st _values = 0.0023; median SD = 0.0014; mean coefficient of variance for all F_st _values = 0.0023) indicate that this approach resulted in good estimations of paired F_st _values.

The 93 SNP AIM subset had substantially larger intercontinental paired F_st _values than the random SNPs for any of the pairs of population groups from the sub-Saharan African, Amerindian, East Asian, Oceana and European (excluding the South Asian populations) continental groups (Table 1). For the South Asian populations, the paired F_st _values showed a similar pattern, with the exception of those between the South Asian and Oceana groups in which the paired F_st _values using the AIM panel were similar to those determined using the random SNPs. In contrast, the paired F_st _values within continental groups (European, Amerindian, East Asia, and Oceana) were very similar when comparing the AIM and random SNP sets (mean intra-continental group F_st _difference = 0.008). Overall, the F_st _values determined using the 93 AIM set were highly correlated with the F_st _values determined using the random SNPs (r^2 ^= 0.70) (Additional file 2).

**Table 1:** Paired F_st _values using 93 AIMs and random sets of 3500 SNPs^a^

	**CHB**	**YAK**	**FIL**	**AJA**	**IRISH**	**SWED**	**PAL**	**MAYA**	**COL**	**PYG**	**YRI**	**BAL**	**BUR**	**KAL**	**UYG**	**MEL**	**PAP**
**CHB^b^**		0.040	0.012	0.260	0.311	0.310	0.246	0.223	0.191	0.461	0.470	0.176	0.142	0.221	0.074	0.147	0.186
**YAK**	0.029		0.040	0.198	0.249	0.247	0.187	0.268	0.224	0.503	0.506	0.118	0.083	0.153	0.041	0.152	0.162
**FIL**	0.014	0.043		0.234	0.285	0.289	0.219	0.248	0.217	0.440	0.452	0.149	0.110	0.199	0.062	0.124	0.153
**AJA**	0.108	0.087	0.106		0.014	0.017	0.014	0.459	0.450	0.487	0.504	0.027	0.061	0.035	0.091	0.250	0.234
**IRISH**	0.112	0.089	0.111	0.011		0.007	0.033	0.492	0.493	0.520	0.539	0.059	0.096	0.069	0.129	0.308	0.294
**SWED**	0.109	0.087	0.108	0.012	0.002		0.030	0.492	0.492	0.527	0.467	0.069	0.102	0.066	0.129	0.317	0.302
**PAL**	0.111	0.091	0.108	0.010	0.019	0.020		0.459	0.443	0.467	0.484	0.022	0.051	0.051	0.084	0.240	0.236
**MAYA**	0.109	0.104	0.119	0.133	0.131	0.128	0.140		0.030	0.598	0.602	0.398	0.380	0.432	0.308	0.408	0.415
**COL**	0.125	0.120	0.137	0.146	0.143	0.141	0.152	0.035		0.672	0.661	0.373	0.352	0.420	0.268	0.407	0.415
**PYG**	0.217	0.214	0.221	0.173	0.184	0.182	0.160	0.260	0.276		0.031	0.471	0.503	0.511	0.496	0.481	0.497
**YRI**	0.191	0.186	0.192	0.146	0.159	0.156	0.133	0.232	0.247	0.048		0.483	0.497	0.522	0.504	0.477	0.495
**BAL**	0.093	0.074	0.091	0.018	0.021	0.021	0.016	0.125	0.136	0.168	0.138		0.013	0.014	0.040	0.176	0.169
**BUR**	0.082	0.065	0.083	0.027	0.030	0.029	0.027	0.122	0.136	0.183	0.152	0.008		0.041	0.017	0.170	0.172
**KAL**	0.116	0.100	0.116	0.047	0.046	0.044	0.049	0.145	0.159	0.201	0.172	0.035	0.040		0.065	0.233	0.226
**UYG**	0.035	0.028	0.042	0.035	0.035	0.034	0.036	0.095	0.109	0.182	0.152	0.021	0.017	0.049		0.141	0.158
**MEL**	0.139	0.149	0.140	0.153	0.157	0.155	0.156	0.208	0.232	0.259	0.232	0.146	0.146	0.169	0.130		0.088
**PAP**	0.170	0.177	0.175	0.169	0.173	0.170	0.172	0.228	0.258	0.271	0.245	0.164	0.168	0.186	0.155	0.105	

a. The Paired Fst value were determined using the Weir and Cockerham algorithm [9]. Above the diagonal of identity the F_st _values were determined using the 93 SNP AIMs. Below the diagonal is the mean determined from three nonoverlapping sets of 3500 SNPs.

b. Population group abreviations included Chinese from Beijing (CHB) from HapMap data, Yakut (YAK), Filipino (FIL), Ashkenazi American (AJA), Swedish (SWED), Maya (MAYA), Palestinian (PAL), Columbian (COL), Mbuti Pygmy (PYG), YRI (Yorubon, HapMap data), Balochi (BAL), Burusho (BUR), Kalash (KAL), Uygur (UYG), Melanesian (MEL) and Papuan (PAP).

### Examination of Population Structure Using Non-Hierachical Clustering

**Figure 1:**
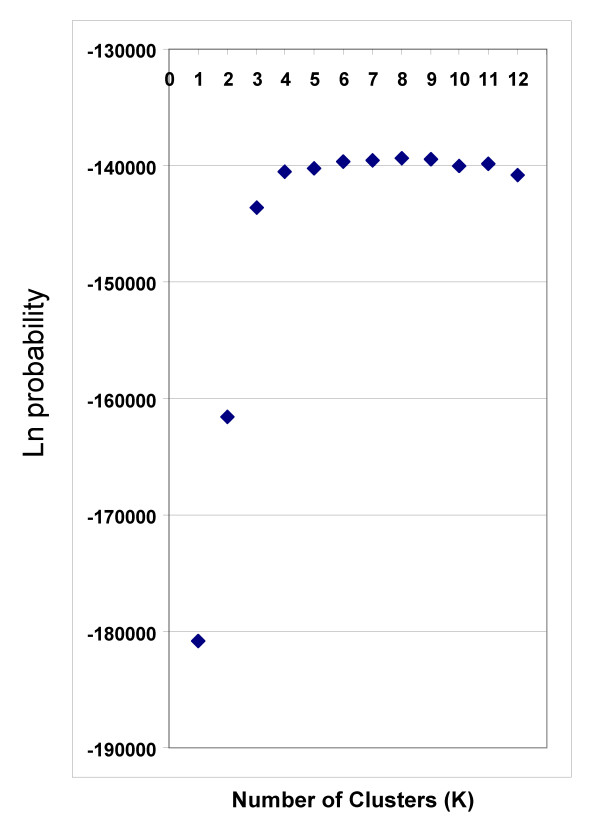
**Probability estimations for the number of cluster groups (K) using STRUCTURE**. The ordinate show the Ln probability corresponding to the number of cluster (K). STRUCTURE analyses were performed using the F model (admixture) as described in **Methods **using the 93 SNP AIM set. The Ln probability closest to zero corresponds to the most likely number of clusters or population groups that explain the population structure.

**Figure 2:**
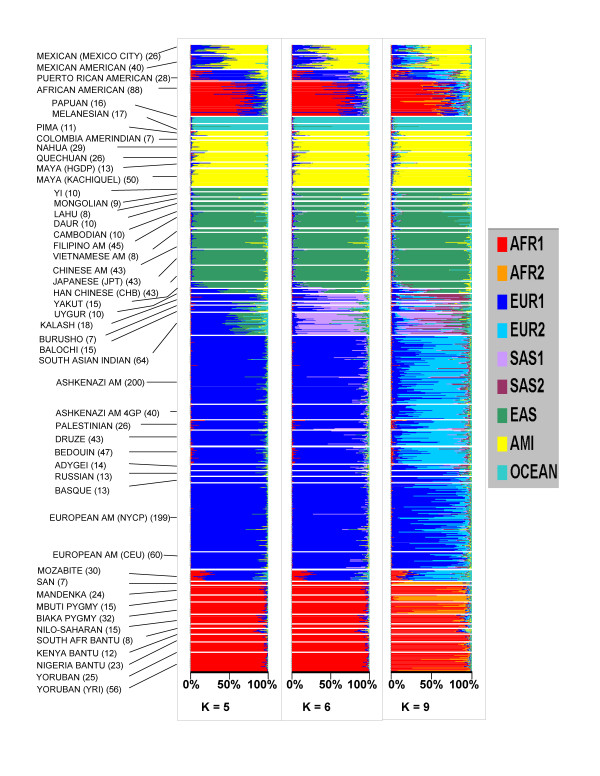
**Analysis of population genetic structure using 93 SNP AIMs**. Each horizontal line represents an individual subject. Each self identified population group is shown along the ordinate. Analyses were performed using STRUCTURE without any prior population assignment (see **Methods**). The number of cluster groups is shown for each panel. The color code corresponds to individual cluster groups that were named according to the continental group with the largest membership in that group.

The population genetic structure of 1620 subjects was examined using the STRUCTURE program [10,11] that applies a Bayesian non-hierarchical clustering method. The genotypes were from 795 new subjects not previously studied, and 825 subjects from our previous studies [4]. All subjects were examined under different assumptions of the number of population groups (clusters) ranging from one to twelve (K = 1, K = 2 ... K = 12) without any pre-assignment of population affiliation. The estimation of Ln probability of the data modestly favored the assumption of K = 9 (Fig 1) and strongly suggested that more than 5 population groups best fit these data. As shown in Fig 2, the population groups corresponded to different self-identified ethnic groupings of specific continental origins and particular sub-continental groupings. When large numbers of replicates were used (see **Methods**), multiple runs of this data set showed stable results at K = 5 and K = 6. When larger numbers of groups were assumed (i.e. K > 6) there was variation in the results. In particular runs various cluster groups would be present or absent. These included some runs in which Bedouin subjects corresponded to an individual cluster group, and others in which the South Asian populations was represented as a single cluster rather than two clusters as shown for K = 9 in Fig 2. Consistent with our previous studies, we observed one or more clusters that showed a high proportion in South Asian population groups and low membership of all other ethnic groups. Interestingly, we also consistently observed a splitting of European populations into two or more clusters that appears to correlate with a distinction between individuals of northern European ancestry and those of ethic groups derived from the Middle-East region. Thus, Palestinian, Bedouin, Druze, and Ashkenazi populations had many individuals with a large membership in a second European cluster (Fig 2, K = 9). Similarly, a division within the sub-Saharan African populations was observed with the majority of San and Mbuti Pygmy individuals showing a high proportion of membership in a second sub-Saharan African cluster. The sub-Saharan Africa results are consistent with observations in a variety of previous studies [5,15-17].

**Figure 3:**
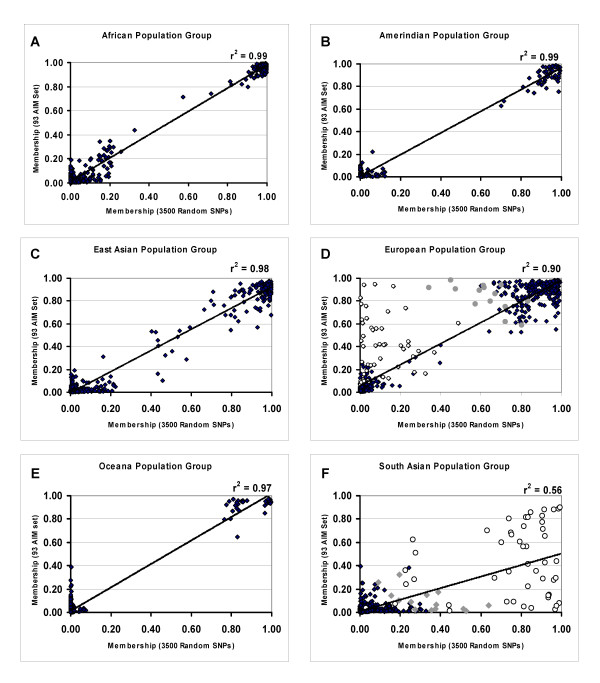
**Correlations between population structure results**. Results of STRUCTURE analyses using the 93 SNP AIM Set and 3500 random SNPs are shown. The fraction of membership for each individual analyzed is indicated for each of six clusters (panels **A**-**F**) for the 93 SNP AIM set (ordinate) and a 3500 SNP set (abscissa). The population clusters named according to the continental group with the largest membership in that group. The subjects included each of the HGDP, HapMap and the Maya (Kachiquel) individuals (see methods). The results show a single 3500 random SNP set is shown. However each of three independent 3500 random SNP sets show very similar results. For panels **D **and **F**, the South Asian ethnic groups (open circles) and Adygei ethnic group (grey circles) are shown with different symbols to highlight the differences between the 93 SNP AIM set and 3500 random SNPs.

The STRUCTURE analyses using the 93 SNP AIM set were also compared with results obtained using random sets of 3500 SNPs for K = 6 (Fig 3). In this comparison we used HGDP, HapMap and Maya (Kachiquel) samples. The individual membership in each cluster group was highly correlated: overall r^2 ^= 0.94; for the African cluster, r^2 ^= 0.99; for the Amerindian cluster, r^2 ^= 0.99; for the East Asian cluster, r^2 ^= 0.99; for the European cluster, r^2 ^= 0.90; for the South Asian cluster, r^2 ^= 0.56; and for the Oceana cluster, r^2 ^= 0.97. The weakest correlations were observed for the Burusho, Balochi, and Kalash South Asian ethnic groups, and the Adygei individuals. For these particular ethnic groups the membership in the European and South Asian groups was substantially different using the 93 SNP AIM set than the result obtained using 3500 random SNPs (Fig. 3D and 3F).

There was also a correlation between the 93 AIM set and 3500 random SNP set when the splitting of the European and Sub-Saharan African populations was observed (e.g. when K = 9 analyses were performed). The correlation in membership in the two European clusters (r^2 ^= 0.50) and two sub-Saharan African clusters (r^2 ^= 0.44) provides support for the ability of the 93 SNP AIM set to partially discern these additional aspects of population substructure.

The performance of the 93 SNP AIM set was also compared with results obtained using different numbers of random SNPs. Overall, the 93 SNP AIM set showed marginally higher correlations with group membership determined using 3500 random SNPs (r^2 ^= 0.94), than did random sets of 500 SNPs (r^2 ^= 0.90). The performance of the 93 SNP AIM set was also examined using restricted sample groups (European, sub-Saharan African and Mozabites) to assess the European and sub-Saharan African contribution in the Mozabite ethnic group. For this comparison, the STRUCTURE analyses were performed using K = 2. Here, the correlation between the 93 SNP AIM set and 3500 random SNPs was stronger than the correlation observed for 500 random SNP sets and nearly comparable to 1000 random SNPs. The 93 SNP AIM set showed an r^2 ^= 0.85 with a 3500 random SNP set. Each of three independent random 500 SNP sets showed lower r^2 ^values with the 3500 random SNPs (r^2 ^values = 0.64, 0.75 and 0.77, respectively, for three independent 500 SNP sets). For sets of 1000 random SNPs, very high correlations were observed (r^2 ^= 0.90, 0.87, and 0.91 for three independent random 1000 SNP sets). As a comparison, 93 random SNP sets showed much lower correlations (r^2 ^values = 0.44, 0.27, and 0.36 for three independent random SNP sets). Together, these data suggest that the 93 SNP AIMs capture more ancestry information than random sets of 500 SNPs and are nearly comparable to using 1000 random SNPs.

To further assess the correspondence of the cluster groups to geographic ancestry, we examined the K = 6 STRUCTURE results comparing the presumed European, East Asian, sub-Saharan African, Oceana, and Amerindian population clusters with each of the self identified or regionally collected groups (Table 2). For the purposes of these analyses, we considered South Asian origin as a distinct group separate from "European" populations (see **Discussion**). Using >0.85 membership in a cluster as the criterion for inclusion, most of the subjects within each self-defined or collected population group corresponded to the expected continental group (Table 2). For example, of the European subjects 91.9% were members of the "European" cluster group and none were members of the other five cluster groups.

**Table 2:** Ascertainment of Continental Ancestry Using 93 SNP AIM Panel

	**STRUCTURE Criterion^a^**					**Eigenvector Criterion**				
	**EUR**	**EAS**	**AFR**	**OCEAN**	**AMI**	**EUR**	**EAS**	**AFR**	**OCEAN**	**AMI**
**European **(643)^b^	**91.9%**	**0.0%**	**0.0%**	**0.0%**	**0.0%**	**87.1%**	**0.0%**	**0.0%**	**0.0%**	**0.0%**
Ashkenazi (40)	97.5%	0.0%	0.0%	0.0%	0.0%	95.0%	0.0%	0.0%	0.0%	0.0%
Palestinian (26)	73.1%	0.0%	0.0%	0.0%	0.0%	80.8%	0.0%	0.0%	0.0%	0.0%
Bedouin (47)	78.7%	0.0%	0.0%	0.0%	0.0%	80.9%	0.0%	0.0%	0.0%	0.0%
Russian (13)	92.3%	0.0%	0.0%	0.0%	0.0%	84.6%	0.0%	0.0%	0.0%	0.0%
CEU (48)	93.8%	0.0%	0.0%	0.0%	0.0%	93.8%	0.0%	0.0%	0.0%	0.0%
EURA (399)	94.2%	0.0%	0.0%	0.0%	0.0%	88.2%	0.0%	0.0%	0.0%	0.0%
OEUR (70)^c^	90.0%	0.0%	0.0%	0.0%	0.0%	78.6%	0.0%	0.0%	0.0%	0.0%
**East Asian (250)**	**0.0%**	**88.5%**	**0.0%**	**0.0%**	**0.0%**	**0.0%**	**90.6%**	**0.0%**	**0.0%**	**0.0%**
CHB (43)	0.0%	97.7%	0.0%	0.0%	0.0%	0.0%	88.4%	0.0%	0.0%	0.0%
JPT (43)	0.0%	93.0%	0.0%	0.0%	0.0%	0.0%	88.4%	0.0%	0.0%	0.0%
Chinese American (44)	0.0%	84.1%	0.0%	0.0%	0.0%	0.0%	84.1%	0.0%	0.0%	0.0%
Yakut (15)	0.0%	20.0%	0.0%	0.0%	0.0%	0.0%	66.7%	0.0%	0.0%	0.0%
Mongolian (9)	0.0%	88.9%	0.0%	0.0%	0.0%	0.0%	77.8%	0.0%	0.0%	0.0%
Filipino American (42)	0.0%	73.8%	0.0%	0.0%	0.0%	0.0%	88.1%	0.0%	0.0%	0.0%
OEAS (54)	0.0%	64.8%	0.0%	0.0%	0.0%	0.0%	85.2%	0.0%	0.0%	0.0%
**Sub-Saharan AFR (221)**	**0.0%**	**0.0%**	**99.1%**	**0.0%**	**0.0%**	**0.0%**	**0.0%**	**87.3%**	**0.0%**	**0.0%**
YRI (56)	0.0%	0.0%	100.0%	0.0%	0.0%	0.0%	0.0%	87.5%	0.0%	0.0%
Nilo-Saharan (23)	0.0%	0.0%	95.7%	0.0%	0.0%	0.0%	0.0%	91.3%	0.0%	0.0%
Mbuti (15)	0.0%	0.0%	100.0%	0.0%	0.0%	0.0%	0.0%	80.0%	0.0%	0.0%
Biaki (32)	0.0%	0.0%	100.0%	0.0%	0.0%	0.0%	0.0%	100.0%	0.0%	0.0%
Mandeka (24)	0.0%	0.0%	100.0%	0.0%	0.0%	0.0%	0.0%	100.0%	0.0%	0.0%
OSSAFR (71)	0.0%	0.0%	98.6%	0.0%	0.0%	0.0%	0.0%	94.4%	0.0%	0.0%
**South Asian (104)**	**7.7%**	**0.0%**	**0.0%**	**0.0%**	**0.0%**	**18.3%**	**0.0%**	**0.0%**	**0.0%**	**0.0%**
Indian (64)	1.6%	0.0%	0.0%	0.0%	0.0%	9.4%	0.0%	0.0%	0.0%	0.0%
Burusho (7)	0.0%	0.0%	0.0%	0.0%	0.0%	14.3%	0.0%	0.0%	0.0%	0.0%
Balochi (15)	13.3%	0.0%	0.0%	0.0%	0.0%	20.0%	0.0%	0.0%	0.0%	0.0%
Kalash (18)	27.8%	0.0%	0.0%	0.0%	0.0%	50.0%	0.0%	0.0%	0.0%	0.0%
**Amerindian (136)**	**0.0%**	**0.0%**	**0.0%**	**0.0%**	**86.8%**	**0.0%**	**0.0%**	**0.0%**	**0.0%**	**80.1%**
Maya-Kachiquel (50)^c^	0.0%	0.0%	0.0%	0.0%	98.0%	0.0%	0.0%	0.0%	0.0%	98.0%
Maya HGDP (13)	0.0%	0.0%	0.0%	0.0%	46.2%	0.0%	0.0%	0.0%	0.0%	46.2%
Quechuan (26)	0.0%	0.0%	0.0%	0.0%	76.9%	0.0%	0.0%	0.0%	0.0%	88.5%
OAMI (47)	0.0%	0.0%	0.0%	0.0%	91.5%	0.0%	0.0%	0.0%	0.0%	66.0%
**Oceana (32)**	**0.0%**	**0.0%**	**0.0%**	**96.8%**	**0.0%**	**0.0%**	**0.0%**	**0.0%**	**93.5%**	**0.0%**
**OTHER (234)**	**1.7%**	**0.0%**	**9.8%**	**0.0%**	**1.3%**	**2.1%**	**0.0%**	**4.7%**	**0.0%**	**3.8%**
Mozabite (30)	3.3%	0.0%	0.0%	0.0%	0.0%	3.3%	0.0%	0.0%	0.0%	0.0%
Mexican (66)	1.5%	0.0%	0.0%	0.0%	4.5%	1.5%	0.0%	0.0%	0.0%	13.6%
African American (100)	1.0%	0.0%	23.0%	0.0%	0.0%	1.0%	0.0%	11.0%	0.0%	0.0%
Uygur (10)	0.0%	0.0%	0.0%	0.0%	0.0%	0.0%	0.0%	0.0%	0.0%	0.0%
Puerto Rican (28)	3.6%	0.0%	0.0%	0.0%	0.0%	7.1%	0.0%	0.0%	0.0%	0.0%

a. For STRUCTURE, the criterion was >0.85 membership in a particular cluster using K = 6. For PCA the criterion was mean +/- 2 SD for each of the first four PCs where the mean was determined based on the self-identified ethnic group.

b. The continental group is shown in bold and selected individual ethnic groups presented below each continental heading. For presentation purposes many of the individual ethnic groups were placed together: other European (OEUR), other East Asian (OEAS), other sub-Saharan African (OSSAFR) and other Amerindian (OAMI).

c. The Maya-Kachiquel were Maya from the Kachiquel language group as previously described[7] and is from a collection distinct from the HGDP Maya group.

With respect to the South Asian population groups, overall 7.7% (8/104) of the individuals were included in the European group and none of the South Asian individuals were included in any of the other continental categories. Of these eight South Asian individuals included in the European group, five belonged to the Kalash ethnic group. For the South Asian subjects, the criterion of >0.85 membership in a specific cluster included only a minority (27/104) of these subjects. Decreasing the criterion to >0.50 membership resulted in the inclusion of the majority of the South Asian individuals within a single cluster (67/104). Using this criterion, there were only 6 individuals of other ethnic groups that were included in the South Asia group (2/10 Uygur, 1/40 Ashkenazi, 1/26 Palestinian, 1/399 EURA, and 1/43 Druze individuals).

As expected, the vast majority of individuals from admixed populations (African American, Mexican American, and Mexican) were not included in any of the continental groupings. In addition, the Uygur individuals from central Asia and all but one of the Mozabite subjects were excluded from any of the continental groups using the >0.85 cluster group membership criterion.

### Principal Component Analyses of Diverse Population Groups Using AIMs

**Figure 4:**
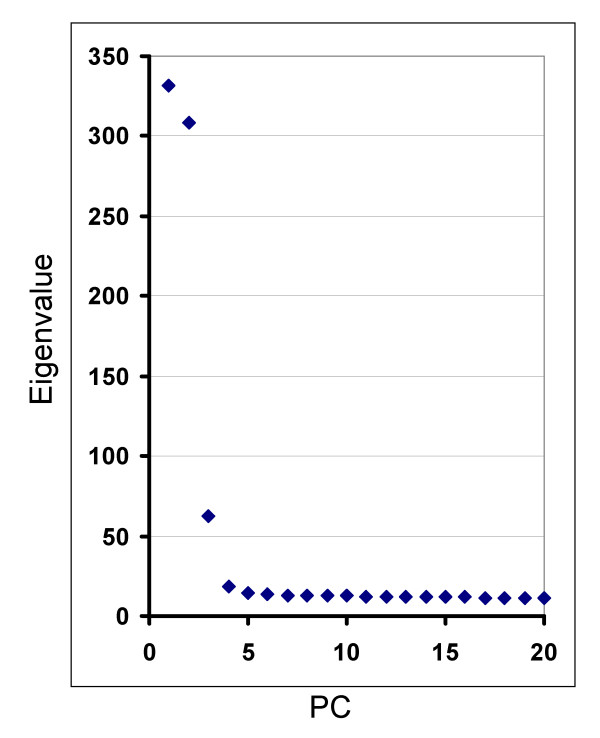
**Eigenvalue distribution for principal components**. The eigenvalues for each PC are shown. Comparing the eigenvalue of each PC shows the relative amount of variation that is explained by the different PCs. The plateau in eigenvalues generally corresponds to variation that can not be attributable to discernable groupings of subjects.

**Figure 5:**
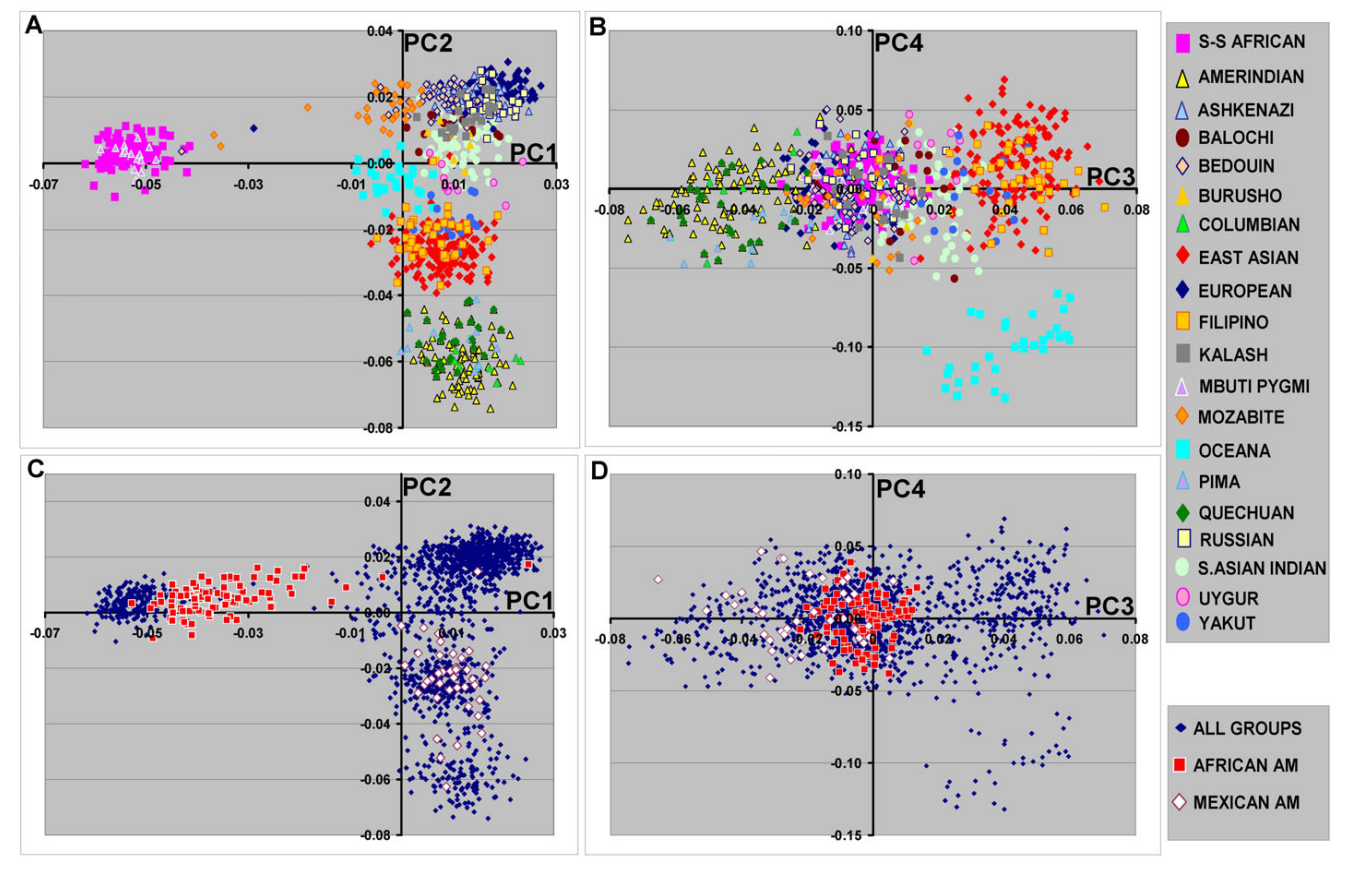
**Principal component analysis of diverse population groups**. The analysis used the same data set indicated in Figure 2. The population groups are shown by the color coded symbols. The sub-Saharan African groups are designated S-S African in this figure. **A**, shows the PC1 and PC2 results from the different ethnic groups excluding the admixed populations. **C**, shows the PC1 and PC2 results for the African American and Mexican American subjects that were run together with the individual subjects shown in **A**. **B, **and **D **show the results for the same subject groups for PC3 and PC4.

The same data set was also examined using principal component analyses (PCA). Nearly all of the variance detected using the 93 AIM set was defined by the first four principal components (PCs) (Fig 4). Similar to the results from STRUCTURE, the first four PCs (Fig 5) show the separation of the 5 continental populations as well as those of two admixed populations (African American and Mexican American groups) that were included in the analyses.

Also similar to the cluster groups defined by STRUCTURE, putative subject groups corresponding to continents or admixed population groups could be assigned using the individual subject eigenvector scores. Here, we used the self-identified or collected European, East Asian, sub-Saharan African, Oceana, and Amerindian populations groups to define the groups. The criterion for inclusion was the mean +/- two standard deviations (SD) of the eigenvector scores for each of the first four PCs (i.e. individuals with eigenvector scores > mean + 2 SD or < mean - 2SD for PC1, PC2, PC3, or PC4 were excluded). Using this definition, most of the subjects within each self-defined or collected population group were included within a self-matching group (Table 2). Similar to the STRUCTURE results, with the exception of a single Mozabite individual, none of the subjects that were a priori considered to be of other continental populations (excluding South Asian and admixed population groups) were included in any of the other continental groups.

For South Asians, this criterion (mean +/- 2 SD) included 87 of the 104 self-identified subjects in the South Asian grouping. However, 170 of the remaining 1516 subjects (not self-identified as South Asian) were also included in this group. When the criterion was changed to the mean +/- 1 SD, only 25 of the 104 self-identified South Asian subjects were included in this group and 8 other non-South Asian subjects were also included. Thus, for the 93 AIM set data, the PCA analyses did not perform well with respect to identifying South Asian subjects.

**Figure 6:**
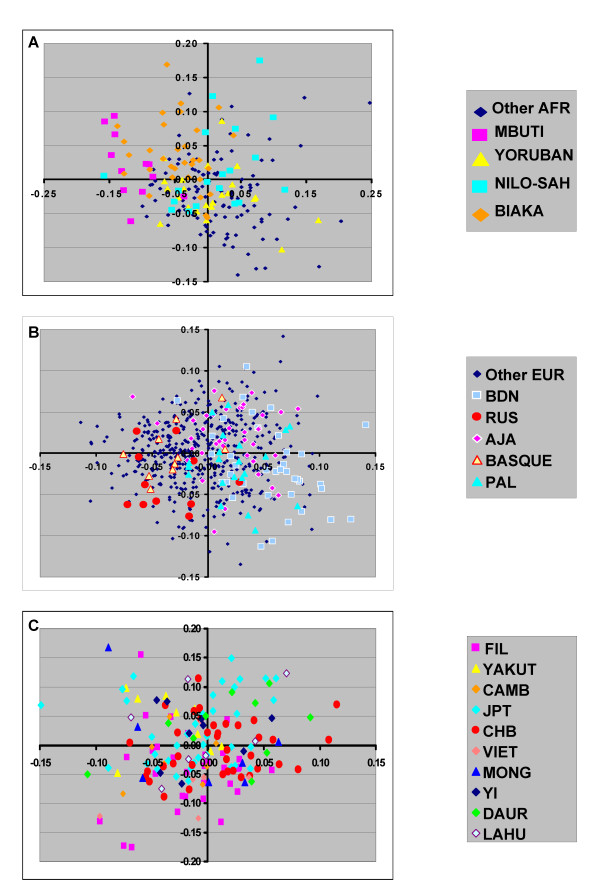
**Principal component analysis of sub-Saharan African, European and East Asian populations**. The analysis was performed using only the individual continental groups. The populations included each of the sub-Saharan African (**A**), European (**B**), and East Asian (**C**) ethnic groups. The color code highlights specific ethnic groups.

Using PCA, we further examined these individual population groups. There was partial grouping of certain ethnic groups when only those subjects within individual continental groups were analyzed separately (Fig 6). This was most distinct for the Mbuti Pygmy group within the sub-Saharan African populations. In addition, southern European population groups could be partially distinguished other European population groups (e.g. Palestinian compared to Russian). However, clustering of different East Asian populations groups was not observed using this set of AIMs selected for continental differences (see **Discussion**).

## Discussion

The current study provides additional validation for the use of a set of AIMs in human genetic studies. We show that a set of 93 SNP AIMs can distinguish a wide variety of diverse population groups in a sampling that includes the most populous groups in the United States as well as many groups from each continent with the exception of Australia. For example, even very diverse tribal groups within Africa can be readily distinguished from other continental groups. In addition, since the AIMs were in large part initially selected to distinguish between Amerindian, European, and African ancestry, the same set of AIMs provides good information for individual admixture in the largest admixed population groups (African American and Mexican American) in the United States. This AIM set also distinguished most of the South Asian and Central Asian individuals from those of European or East Asian origin and was also effective in grouping Oceana populations. Although there were specific limitations (e.g. for distinguishing South Asian populations), overall, the data suggest that this AIM set performs better than 500 random SNPs for distinguishing continental population differences.

Although many previous studies have identified AIMs that distinguish particular combinations of continental groups [2,18-20], the current AIM set has several important features. These include: 1) validation using many different population groups from all continents with the exception of Australia; and 2) widely available genotyping results that can be readily incorporated in analyses. The latter includes the previously published individual genotypes accompanying our initial study of these SNPs [4], and any subject sets genotyped using the Illumina 300 K or larger SNP platforms. Importantly, both the HGDP and HapMap Illumina genotypes are publically available. In fact, the performance of the current AIM set could not be directly compared with other recently described AIM sets [2,21] because of limited public availability of individual subject HGDP genotypes for these SNPs. The use of previous genotyped data sets in analyses can enhance the performance of analyses using either clustering algorithms or PCA, both of which are influenced by the inclusion of different population groups [22]. Finally, the current set of AIMs has been selected for performance on the widely used TaqMan^® ^platform that can be efficiently applied in small laboratory settings and is commercially available as a marker set .

We have previously discussed and provided general guidelines for the application of AIMs [3,4]. In the current study, we have used specific criteria for both STRUCTURE outputs and PCA eigenvector scores to analyze a SNP AIM panel using additional subjects of diverse ethnic group affiliation. Marginally better correspondence with self-identified ethnic affiliation was observed in this data set using the model dependent clustering algorithm applied in STRUCTURE compared to PCA (Table 2). However, PCA may offer substantial computational advantages if the AIMs are used for controlling population structure and substructure in association studies [3,12]. Thus, at present, we would suggest using STRUCTURE results for limiting analyses to particular subject groups and using PCA or multidimensional scaling for association testing. The application of multi-dimensional scaling showed nearly identical results to those using PCA (data not shown).

It is worth noting that this current AIM panel excludes nearly all South Asian subjects from "other" European populations. As has been noted in previous studies, South Asian populations are much closer to European than East Asian or other continental groups and South Asian ethnic populations are variably grouped together with European population groups [23]. When comparing population differentiation using paired F_st _values there is no clear distinction between these different European and South Asian ethnic groups (Table 1). For example, the following F_st _values using random SNPs were observed: Balochi/Ashkenazi = 0.018, Balochi/Palestinian = 0.016, Balochi/Swedish = 0.021, Palestinian/Swedish = 0.020, Palestinian/Ashkenazi = 0.010, Ashkenazi/Swedish = 0.012. However, the current STRUCTURE results that show South Asian specific clusters, previous STRUCTURE analyses [20,23], and PCA analyses using thousands of SNPs [5] indicate substantial differences in the allele patterns of South Asian compared to European subjects. Thus, it may be advantageous to exclude South Asian subjects in European association studies to reduce genetic heterogeneity. The current suggested criteria (Table 2) will probably exclude most South Asian individuals, although with the caveat that many South Asian ethnic groups have not been studied.

This 93 SNP AIM set also showed a partial ability to discern additional population substructure. For both Europeans and sub-Saharan Africans, there was apparent grouping of certain ethnic groups in additional clusters. This was most clear for K = 9 in the STRUCTURE analysis but was also suggested by the graphic representation in the PCA analysis (Fig 6). Thus, the differences between Arab and Ashkenazi European and northern European ethnic groups, and the difference between certain sub-Saharan African groups (e.g. Mbuti Pygmy) are partially discerned. However, previous studies by multiple groups indicate that additional panels of SNPs are necessary to most effectively control for differences in European population substructure [22,24-26]. In addition, the 93 SNP AIM panel did not show any substructure within the East Asian populations. Recent studies using HGDP and other sample sets show substructure within East Asian population groups further emphasizing the potential limitations of the 93 SNP AIM panel [5,27]. The current AIM panel is designed to address continental differences and we caution that controlling for population stratification within particular continental groups requires additional panels of SNPs to further reduce false positive or negative results in association tests [3,22,24-28]. Importantly, the current AIM set performs well with respect to ascertaining admixture proportions in African Americans and in Hispanic populations [4]. The need for utilizing additional SNPs for addressing population stratification will be highly dependent on the populations being used in a particular study and whether other strategies including demographic information are used for matching cases and controls.

## Conclusion

The current study provides additional confidence that a panel of 93 AIMs can be effectively used to ascertain population genetic structure that results from the inclusion of subjects of diverse continental origins. Using either highly supervised clustering algorithms or largely unsupervised PCA, these SNP AIMS can be used to 1) identify continental subject groups for genetic studies, 2) identify study population outliers, and 3) control for admixture in association studies.

## Competing interests

PAW and FMDLV are employees of Applied Biosystems.

## Authors' contributions

The study was conceived by MFS, FMDLV and designed by RN, RKo, and MFS. PKG, MEAR, RKi, LMB, GS, and JWB recruited subjects, and obtained and prepared DNA samples used in these studies. Genotyping was performed by RN and PKG. Analyses were performed by RN, RKo, CT and MFS. The manuscript was written by RN and MFS with contributions from RKo, CT, LMB, PAW, FMDLV and PKG. All authors have read and approved the final manuscript.

## Supplementary Material

Additional file 1 **SNP Ancestry Informative Markers**. List of SNPs including sequence context and chromosomal locations. Click here for file

Additional file 2 **Correlation of F_st _values between 93 SNP AIM set and 3500 random SNPs**. Figure showing the correlation between interpopulation F_st _values calculated using the 93 SNP AIM set compared with the result using random 3500 SNPs (mean from three independent sets). Click here for file
