# Supplementary figures and images for: An ancestry informative marker set for determining continental origin: validation and extension using human genome diversity panels
Source: BMC Genet. 2009 Jul 24;10:39. doi: 10.1186/1471-2156-10-39 (PMC2728728; doi:10.1186/1471-2156-10-39)

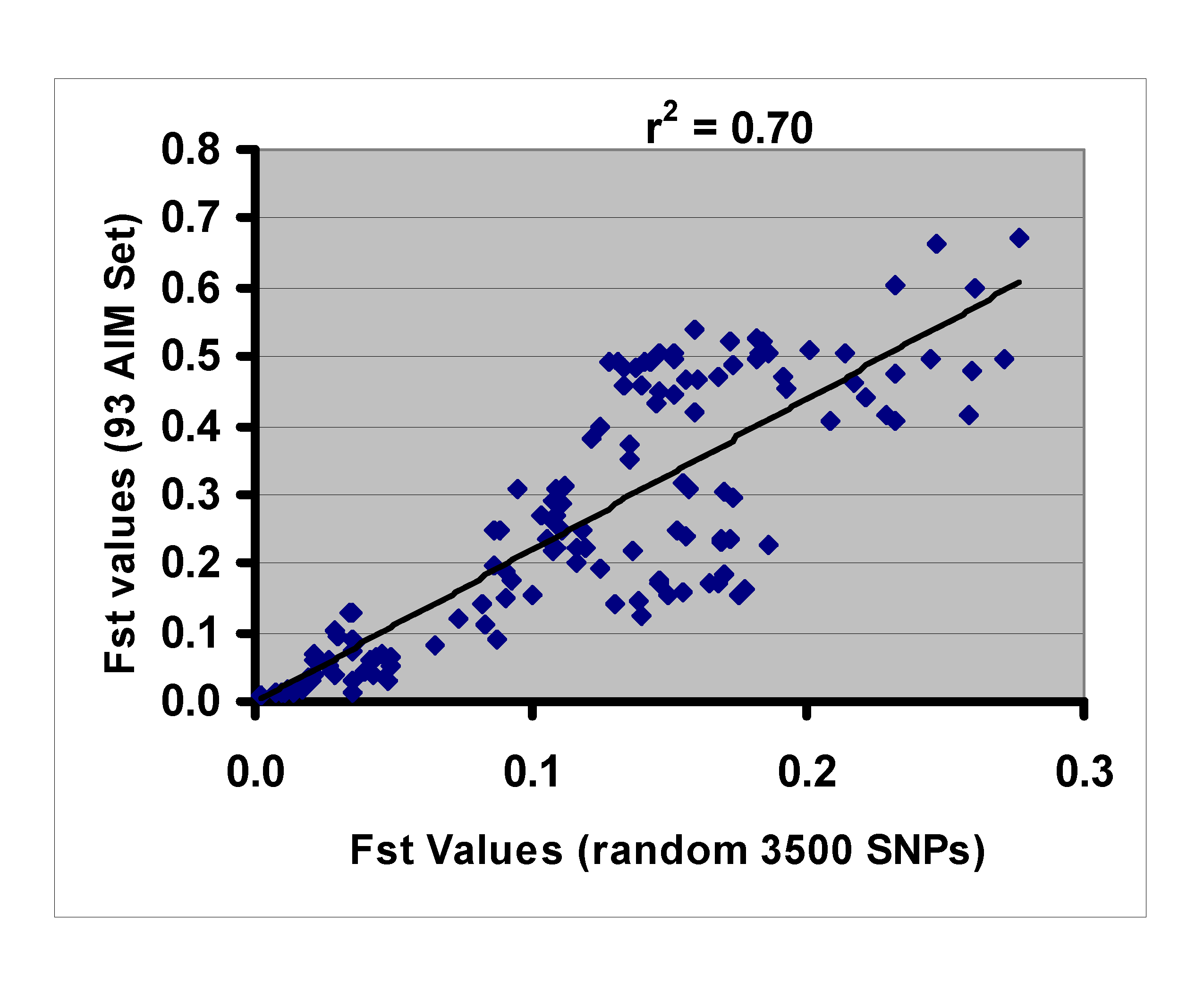

Supplement: Additional file 2 — Correlation of Fst values between 93 SNP AIM set and 3500 random SNPs. Figure showing the correlation between interpopulation Fst values calculated using the 93 SNP AIM set compared with the result using random 3500 SNPs (mean from three independent sets). [file 1471-2156-10-39-S2.tiff]
